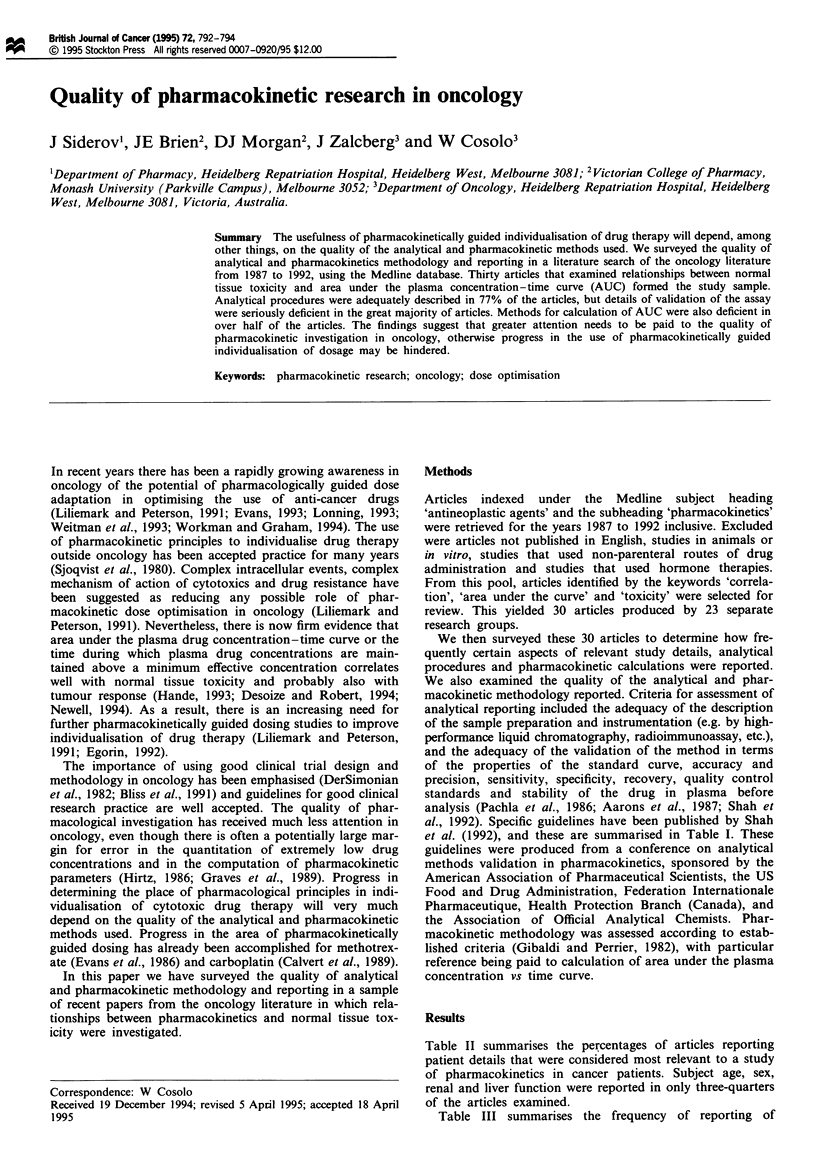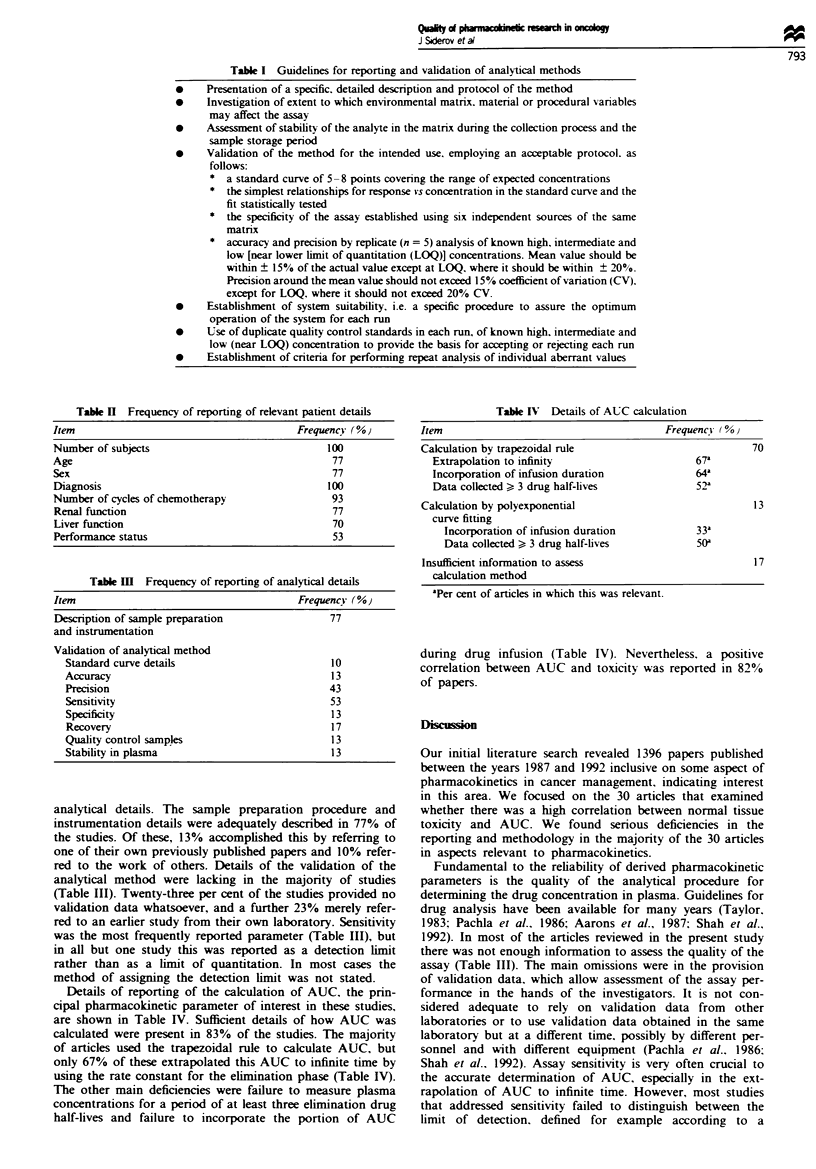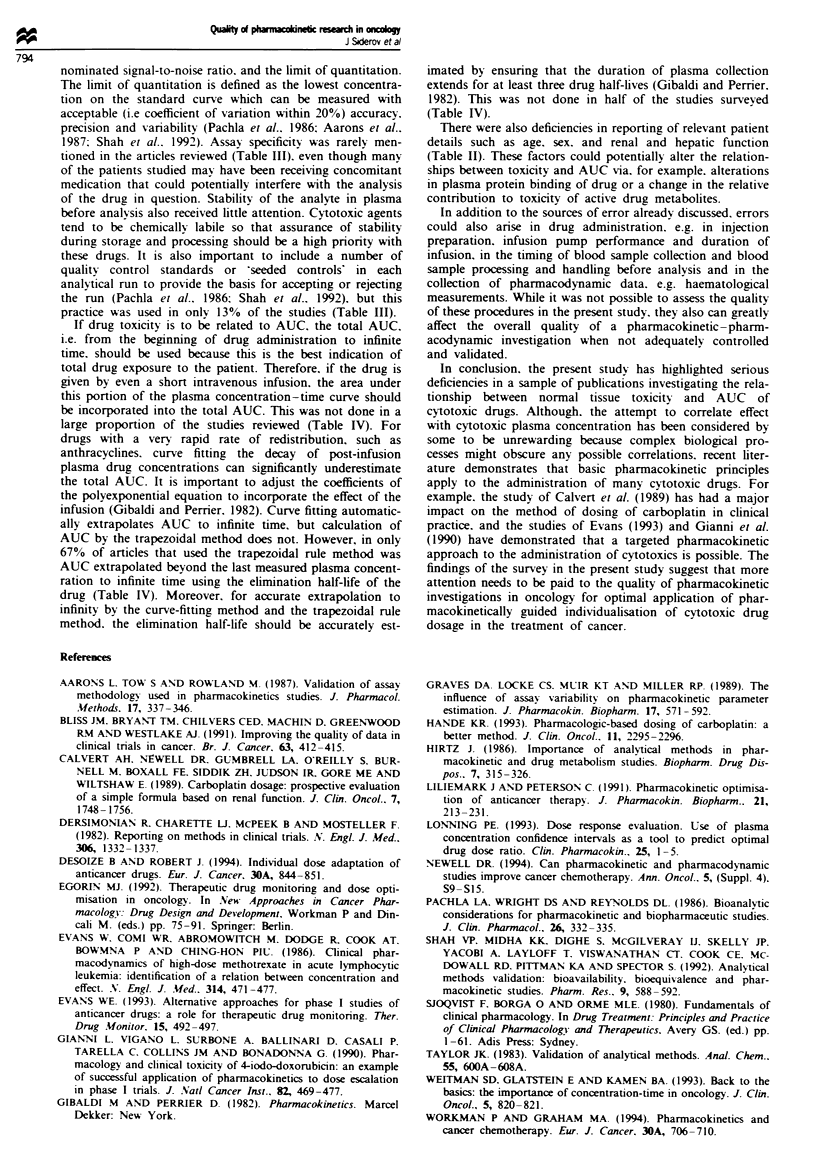# Quality of pharmacokinetic research in oncology.

**DOI:** 10.1038/bjc.1995.413

**Published:** 1995-09

**Authors:** J. Siderov, J. E. Brien, D. J. Morgan, J. Zalcberg, W. Cosolo

**Affiliations:** Department of Pharmacy, Heidelberg Repatriation Hospital, Melbourne, Australia.

## Abstract

The usefulness of pharmacokinetically guided individualisation of drug therapy will depend, among other things, on the quality of the analytical and pharmacokinetic methods used. We surveyed the quality of analytical and pharmacokinetics methodology and reporting in a literature search of the oncology literature from 1987 to 1992, using the Medline database. Thirty articles that examined relationships between normal tissue toxicity and area under the plasma concentration-time curve (AUC) formed the study sample. Analytical procedures were adequately described in 77% of the articles, but details of validation of the assay were seriously deficient in the great majority of articles. Methods for calculation of AUC were also deficient in over half of the articles. The findings suggest that greater attention needs to be paid to the quality of pharmacokinetic investigation in oncology, otherwise progress in the use of pharmacokinetically guided individualisation of dosage may be hindered.


British Journal of Cancer (1995) 72, 792-794
© 1995 Stockton Press All rights reserved 0007-0920/95 $12.00

### Quality of pharmacokinetic research in oncology

J Siderov[1], JE Brien[2], DJ Morgan[2], J Zalcberg[3] and W Cosolo[3]

[1]*Department of Pharmacy, Heidelberg Repatriation Hospital, Heidelberg West, Melbourne 3081;* [2]*Victorian College of Pharmacy, Monash University (Parkville Campus), Melbourne 3052;* [3]*Department of Oncology, Heidelberg Repatriation Hospital, Heidelberg West, Melbourne 3081, Victoria, Australia.*

**Summary** The usefulness of pharmacokinetically guided individualisation of drug therapy will depend, among other things, on the quality of the analytical and pharmacokinetic methods used. We surveyed the quality of analytical and pharmacokinetics methodology and reporting in a literature search of the oncology literature from 1987 to 1992, using the Medline database. Thirty articles that examined relationships between normal tissue toxicity and area under the plasma concentration-time curve (AUC) formed the study sample. Analytical procedures were adequately described in 77% of the articles, but details of validation of the assay were seriously deficient in the great majority of articles. Methods for calculation of AUC were also deficient in over half of the articles. The findings suggest that greater attention needs to be paid to the quality of pharmacokinetic investigation in oncology, otherwise progress in the use of pharmacokinetically guided individualisation of dosage may be hindered.

**Keywords:** pharmacokinetic research; oncology; dose optimisation

In recent years there has been a rapidly growing awareness in oncology of the potential of pharmacologically guided dose adaptation in optimising the use of anti-cancer drugs (Liliemark and Peterson, 1991; Evans, 1993; Lonning, 1993; Weitman *et al.*, 1993; Workman and Graham, 1994). The use of pharmacokinetic principles to individualise drug therapy outside oncology has been accepted practice for many years (Sjoqvist *et al.*, 1980). Complex intracellular events, complex mechanism of action of cytotoxics and drug resistance have been suggested as reducing any possible role of pharmacokinetic dose optimisation in oncology (Liliemark and Peterson, 1991). Nevertheless, there is now firm evidence that area under the plasma drug concentration-time curve or the time during which plasma drug concentrations are maintained above a minimum effective concentration correlates well with normal tissue toxicity and probably also with tumour response (Hande, 1993; Desoize and Robert, 1994; Newell, 1994). As a result, there is an increasing need for further pharmacokinetically guided dosing studies to improve individualisation of drug therapy (Liliemark and Peterson, 1991; Egorin, 1992).

The importance of using good clinical trial design and methodology in oncology has been emphasised (DerSimonian *et al.*, 1982; Bliss *et al.*, 1991) and guidelines for good clinical research practice are well accepted. The quality of pharmacological investigation has received much less attention in oncology, even though there is often a potentially large margin for error in the quantitation of extremely low drug concentrations and in the computation of pharmacokinetic parameters (Hirtz, 1986; Graves *et al.*, 1989). Progress in determining the place of pharmacological principles in individualisation of cytotoxic drug therapy will very much depend on the quality of the analytical and pharmacokinetic methods used. Progress in the area of pharmacokinetically guided dosing has already been accomplished for methotrexate (Evans *et al.*, 1986) and carboplatin (Calvert *et al.*, 1989).

In this paper we have surveyed the quality of analytical and pharmacokinetic methodology and reporting in a sample of recent papers from the oncology literature in which relationships between pharmacokinetics and normal tissue toxicity were investigated.

Correspondence: W Cosolo
Received 19 December 1994; revised 5 April 1995; accepted 18 April 1995

### Methods

Articles indexed under the Medline subject heading 'antineoplastic agents' and the subheading 'pharmacokinetics' were retrieved for the years 1987 to 1992 inclusive. Excluded were articles not published in English, studies in animals or *in vitro*, studies that used non-parenteral routes of drug administration and studies that used hormone therapies. From this pool, articles identified by the keywords 'correlation', 'area under the curve' and 'toxicity' were selected for review. This yielded 30 articles produced by 23 separate research groups.

We then surveyed these 30 articles to determine how frequently certain aspects of relevant study details, analytical procedures and pharmacokinetic calculations were reported. We also examined the quality of the analytical and pharmacokinetic methodology reported. Criteria for assessment of analytical reporting included the adequacy of the description of the sample preparation and instrumentation (e.g. by high-performance liquid chromatography, radioimmunoassay, etc.), and the adequacy of the validation of the method in terms of the properties of the standard curve, accuracy and precision, sensitivity, specificity, recovery, quality control standards and stability of the drug in plasma before analysis (Pachla *et al.*, 1986; Aarons *et al.*, 1987; Shah *et al.*, 1992). Specific guidelines have been published by Shah *et al.* (1992), and these are summarised in Table I. These guidelines were produced from a conference on analytical methods validation in pharmacokinetics, sponsored by the American Association of Pharmaceutical Scientists, the US Food and Drug Administration, Federation Internationale Pharmaceutique, Health Protection Branch (Canada), and the Association of Official Analytical Chemists. Pharmacokinetic methodology was assessed according to established criteria (Gibaldi and Perrier, 1982), with particular reference being paid to calculation of area under the plasma concentration *vs* time curve.

### Results

Table II summarises the percentages of articles reporting patient details that were considered most relevant to a study of pharmacokinetics in cancer patients. Subject age, sex, renal and liver function were reported in only three-quarters of the articles examined.

Table III summarises the frequency of reporting of

**Table I** Guidelines for reporting and validation of analytical methods

- Presentation of a specific, detailed description and protocol of the method
- Investigation of extent to which environmental matrix, material or procedural variables may affect the assay
- Assessment of stability of the analyte in the matrix during the collection process and the sample storage period
- Validation of the method for the intended use, employing an acceptable protocol, as follows:
  * a standard curve of 5–8 points covering the range of expected concentrations
  * the simplest relationships for response *vs* concentration in the standard curve and the fit statistically tested
  * the specificity of the assay established using six independent sources of the same matrix
  * accuracy and precision by replicate ($n = 5$) analysis of known high, intermediate and low [near lower limit of quantitation (LOQ)] concentrations. Mean value should be within ± 15% of the actual value except at LOQ, where it should be within ± 20%. Precision around the mean value should not exceed 15% coefficient of variation (CV), except for LOQ, where it should not exceed 20% CV.
- Establishment of system suitability, i.e. a specific procedure to assure the optimum operation of the system for each run
- Use of duplicate quality control standards in each run, of known high, intermediate and low (near LOQ) concentration to provide the basis for accepting or rejecting each run
- Establishment of criteria for performing repeat analysis of individual aberrant values

**Table II** Frequency of reporting of relevant patient details

| *Item* | *Frequency (%)* |
|---|---|
| Number of subjects | 100 |
| Age | 77 |
| Sex | 77 |
| Diagnosis | 100 |
| Number of cycles of chemotherapy | 93 |
| Renal function | 77 |
| Liver function | 70 |
| Performance status | 53 |

**Table III** Frequency of reporting of analytical details

| *Item* | *Frequency (%)* |
|---|---|
| Description of sample preparation and instrumentation | 77 |
| Validation of analytical method | |
| Standard curve details | 10 |
| Accuracy | 13 |
| Precision | 43 |
| Sensitivity | 53 |
| Specificity | 13 |
| Recovery | 17 |
| Quality control samples | 13 |
| Stability in plasma | 13 |

**Table IV** Details of AUC calculation

| *Item* | *Frequency (%)* | |
|---|---|---|
| Calculation by trapezoidal rule | | 70 |
| Extrapolation to infinity | 67[a] | |
| Incorporation of infusion duration | 64[a] | |
| Data collected ⩾ 3 drug half-lives | 52[a] | |
| Calculation by polyexponential curve fitting | | 13 |
| Incorporation of infusion duration | 33[a] | |
| Data collected ⩾ 3 drug half-lives | 50[a] | |
| Insufficient information to assess calculation method | | 17 |

[a]Per cent of articles in which this was relevant.

analytical details. The sample preparation procedure and instrumentation details were adequately described in 77% of the studies. Of these, 13% accomplished this by referring to one of their own previously published papers and 10% referred to the work of others. Details of the validation of the analytical method were lacking in the majority of studies (Table III). Twenty-three per cent of the studies provided no validation data whatsoever, and a further 23% merely referred to an earlier study from their own laboratory. Sensitivity was the most frequently reported parameter (Table III), but in all but one study this was reported as a detection limit rather than as a limit of quantitation. In most cases the method of assigning the detection limit was not stated.

Details of reporting of the calculation of AUC, the principal pharmacokinetic parameter of interest in these studies, are shown in Table IV. Sufficient details of how AUC was calculated were present in 83% of the studies. The majority of articles used the trapezoidal rule to calculate AUC, but only 67% of these extrapolated this AUC to infinite time by using the rate constant for the elimination phase (Table IV). The other main deficiencies were failure to measure plasma concentrations for a period of at least three elimination drug half-lives and failure to incorporate the portion of AUC during drug infusion (Table IV). Nevertheless, a positive correlation between AUC and toxicity was reported in 82% of papers.

### Discussion

Our initial literature search revealed 1396 papers published between the years 1987 and 1992 inclusive on some aspect of pharmacokinetics in cancer management, indicating interest in this area. We focused on the 30 articles that examined whether there was a high correlation between normal tissue toxicity and AUC. We found serious deficiencies in the reporting and methodology in the majority of the 30 articles in aspects relevant to pharmacokinetics.

Fundamental to the reliability of derived pharmacokinetic parameters is the quality of the analytical procedure for determining the drug concentration in plasma. Guidelines for drug analysis have been available for many years (Taylor, 1983; Pachla *et al.*, 1986; Aarons *et al.*, 1987; Shah *et al.*, 1992). In most of the articles reviewed in the present study there was not enough information to assess the quality of the assay (Table III). The main omissions were in the provision of validation data, which allow assessment of the assay performance in the hands of the investigators. It is not considered adequate to rely on validation data from other laboratories or to use validation data obtained in the same laboratory but at a different time, possibly by different personnel and with different equipment (Pachla *et al.*, 1986; Shah *et al.*, 1992). Assay sensitivity is very often crucial to the accurate determination of AUC, especially in the extrapolation of AUC to infinite time. However, most studies that addressed sensitivity failed to distinguish between the limit of detection, defined for example according to a nominated signal-to-noise ratio, and the limit of quantitation. The limit of quantitation is defined as the lowest concentration on the standard curve which can be measured with acceptable (i.e coefficient of variation within 20%) accuracy, precision and variability (Pachla *et al.*, 1986; Aarons *et al.*, 1987; Shah *et al.*, 1992). Assay specificity was rarely mentioned in the articles reviewed (Table III), even though many of the patients studied may have been receiving concomitant medication that could potentially interfere with the analysis of the drug in question. Stability of the analyte in plasma before analysis also received little attention. Cytotoxic agents tend to be chemically labile so that assurance of stability during storage and processing should be a high priority with these drugs. It is also important to include a number of quality control standards or 'seeded controls' in each analytical run to provide the basis for accepting or rejecting the run (Pachla *et al.*, 1986; Shah *et al.*, 1992), but this practice was used in only 13% of the studies (Table III).

If drug toxicity is to be related to AUC, the total AUC, i.e. from the beginning of drug administration to infinite time, should be used because this is the best indication of total drug exposure to the patient. Therefore, if the drug is given by even a short intravenous infusion, the area under this portion of the plasma concentration-time curve should be incorporated into the total AUC. This was not done in a large proportion of the studies reviewed (Table IV). For drugs with a very rapid rate of redistribution, such as anthracyclines, curve fitting the decay of post-infusion plasma drug concentrations can significantly underestimate the total AUC. It is important to adjust the coefficients of the polyexponential equation to incorporate the effect of the infusion (Gibaldi and Perrier, 1982). Curve fitting automatically extrapolates AUC to infinite time, but calculation of AUC by the trapezoidal method does not. However, in only 67% of articles that used the trapezoidal rule method was AUC extrapolated beyond the last measured plasma concentration to infinite time using the elimination half-life of the drug (Table IV). Moreover, for accurate extrapolation to infinity by the curve-fitting method and the trapezoidal rule method, the elimination half-life should be accurately estimated by ensuring that the duration of plasma collection extends for at least three drug half-lives (Gibaldi and Perrier, 1982). This was not done in half of the studies surveyed (Table IV).

There were also deficiencies in reporting of relevant patient details such as age, sex, and renal and hepatic function (Table II). These factors could potentially alter the relationships between toxicity and AUC via, for example, alterations in plasma protein binding of drug or a change in the relative contribution to toxicity of active drug metabolites.

In addition to the sources of error already discussed, errors could also arise in drug administration, e.g. in injection preparation, infusion pump performance and duration of infusion, in the timing of blood sample collection and blood sample processing and handling before analysis and in the collection of pharmacodynamic data, e.g. haematological measurements. While it was not possible to assess the quality of these procedures in the present study, they also can greatly affect the overall quality of a pharmacokinetic-pharmacodynamic investigation when not adequately controlled and validated.

In conclusion, the present study has highlighted serious deficiencies in a sample of publications investigating the relationship between normal tissue toxicity and AUC of cytotoxic drugs. Although, the attempt to correlate effect with cytotoxic plasma concentration has been considered by some to be unrewarding because complex biological processes might obscure any possible correlations, recent literature demonstrates that basic pharmacokinetic principles apply to the administration of many cytotoxic drugs. For example, the study of Calvert *et al.* (1989) has had a major impact on the method of dosing of carboplatin in clinical practice, and the studies of Evans (1993) and Gianni *et al.* (1990) have demonstrated that a targeted pharmacokinetic approach to the administration of cytotoxics is possible. The findings of the survey in the present study suggest that more attention needs to be paid to the quality of pharmacokinetic investigations in oncology for optimal application of pharmacokinetically guided individualisation of cytotoxic drug dosage in the treatment of cancer.

### References

AARONS L, TOWS S AND ROWLAND M. (1987). Validation of assay methodology used in pharmacokinetics studies. *J. Pharmacol. Methods*, **17**, 337-346.

BLISS JM, BRYANT TM, CHILVERS CED, MACHIN D, GREENWOOD RM AND WESTLAKE AJ. (1991). Improving the quality of data in clinical trials in cancer. *Br. J. Cancer*, **63**, 412-415.

CALVERT AH, NEWELL DR, GUMBRELL LA, O'REILLY S, BURNELL M, BOXALL FE, SIDDIK ZH, JUDSON IR, GORE ME AND WILTSHAW E. (1989). Carboplatin dosage: prospective evaluation of a simple formula based on renal function. *J. Clin. Oncol.*, **7**, 1748-1756.

DERSIMONIAN R, CHARETTE LJ, McPEEK B AND MOSTELLER F. (1982). Reporting on methods in clinical trials. *N. Engl. J. Med.*, **306**, 1332-1337.

DESOIZE B AND ROBERT J. (1994). Individual dose adaptation of anticancer drugs. *Eur. J. Cancer*, **30A**, 844-851.

EGORIN MJ. (1992). Therapeutic drug monitoring and dose optimisation in oncology. In *New Approaches in Cancer Pharmacology: Drug Design and Development*, Workman P and Dincali M. (eds.) pp. 75-91. Springer: Berlin.

EVANS W, COMI WR, ABROMOWITCH M, DODGE R, COOK AT, BOWMNA P AND CHING-HON PIU. (1986). Clinical pharmacodynamics of high-dose methotrexate in acute lymphocytic leukemia: identification of a relation between concentration and effect. *N. Engl. J. Med.*, **314**, 471-477.

EVANS WE. (1993). Alternative approaches for phase I studies of anticancer drugs: a role for therapeutic drug monitoring. *Ther. Drug Monitor*, **15**, 492-497.

GIANNI L, VIGANO L, SURBONE A, BALLINARI D, CASALI P, TARELLA C, COLLINS JM AND BONADONNA G. (1990). Pharmacology and clinical toxicity of 4-iodo-doxorubicin: an example of successful application of pharmacokinetics to dose escalation in phase I trials. *J. Natl Cancer Inst.*, **82**, 469-477.

GIBALDI M AND PERRIER D. (1982). *Pharmacokinetics*. Marcel Dekker: New York.

GRAVES DA, LOCKE CS, MUIR KT AND MILLER RP. (1989). The influence of assay variability on pharmacokinetic parameter estimation. *J. Pharmacokin. Biopharm.* **17**, 571-592.

HANDE KR. (1993). Pharmacologic-based dosing of carboplatin: a better method. *J. Clin. Oncol.*, **11**, 2295-2296.

HIRTZ J. (1986). Importance of analytical methods in pharmacokinetic and drug metabolism studies. *Biopharm. Drug Dispos.*, **7**, 315-326.

LILIEMARK J AND PETERSON C. (1991). Pharmacokinetic optimisation of anticancer therapy. *J. Pharmacokin. Biopharm.*, **21**, 213-231.

LONNING PE. (1993). Dose response evaluation. Use of plasma concentration confidence intervals as a tool to predict optimal drug dose ratio. *Clin. Pharmacokin.*, **25**, 1-5.

NEWELL DR. (1994). Can pharmacokinetic and pharmacodynamic studies improve cancer chemotherapy. *Ann. Oncol.*, **5**, (Suppl. 4), S9-S15.

PACHLA LA, WRIGHT DS AND REYNOLDS DL. (1986). Bioanalytic considerations for pharmacokinetic and biopharmaceutic studies. *J. Clin. Pharmacol.*, **26**, 332-335.

SHAH VP, MIDHA KK, DIGHE S, MCGILVERAY IJ, SKELLY JP, YACOBI A, LAYLOFF T, VISWANATHAN CT, COOK CE, McDOWALL RD, PITTMAN KA AND SPECTOR S. (1992). Analytical methods validation: bioavailability, bioequivalence and pharmacokinetic studies. *Pharm. Res.*, **9**, 588-592.

SJOQVIST F, BORGA O AND ORME MLE. (1980). Fundamentals of clinical pharmacology. In *Drug Treatment: Principles and Practice of Clinical Pharmacology and Therapeutics*, Avery GS. (ed.) pp. 1-61. Adis Press: Sydney.

TAYLOR JK. (1983). Validation of analytical methods. *Anal. Chem.*, **55**, 600A-608A.

WEITMAN SD, GLATSTEIN E AND KAMEN BA. (1993). Back to the basics: the importance of concentration-time in oncology. *J. Clin. Oncol.*, **5**, 820-821.

WORKMAN P AND GRAHAM MA. (1994). Pharmacokinetics and cancer chemotherapy. *Eur. J. Cancer*, **30A**, 706-710.